# Surgical Induction of Endolymphatic Hydrops by Obliteration of the Endolymphatic Duct

**DOI:** 10.3791/1728

**Published:** 2010-01-22

**Authors:** Cliff A. Megerian, Chris Heddon, Sami Melki, Suhael Momin, Janis Paulsey, Joy Obokhare, Kumar Alagramam

**Affiliations:** Otolaryngology - Head and Neck Surgery, Case Western Reserve University

## Abstract

Surgical induction of endolymphatic hydrops (ELH) in the guinea pig by obliteration and obstruction of the endolymphatic duct is a well-accepted animal model of the condition and an important correlate for human Meniere's disease.  In 1965, Robert Kimura and Harold Schuknecht first described an intradural approach for obstruction of the endolymphatic duct (Kimura 1965). Although effective, this technique, which requires penetration of the brain's protective covering, incurred an undesirable level of morbidity and mortality in the animal subjects.  Consequently, Andrews and Bohmer developed an extradural approach, which predictably produces fewer of the complications associated with central nervous system (CNS) penetration.(Andrews and Bohmer 1989)

The extradural approach described here first requires a midline incision in the region of the occiput to expose the underlying muscular layer. We operate only on the right side. After appropriate retraction of the overlying tissue, a horizontal incision is made into the musculature of the right occiput to expose the right temporo-occipital suture line.  The bone immediately inferio-lateral the suture line (Fig 1) is then drilled with an otologic drill until the sigmoid sinus becomes visible.  Medial retraction of the sigmoid sinus reveals the operculum of the endolymphatic duct, which houses the endolymphatic sac.  Drilling medial to the operculum into the area of the endolymphatic sac reveals the endolymphatic duct, which is then packed with bone wax to produce obstruction and ultimately ELH.

In the following weeks, the animal will demonstrate the progressive, fluctuating hearing loss and histologic evidence of ELH.

**Figure:**
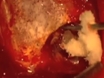


## Protocol

### Part 1: Anesthesia and preparation for surgery

An adult, female Duncan-Hartley guinea pig from Charles River International Laboratories, Inc. (Wilmington, MA) is anesthetized with ketamine 60 mg/kg and xylazine 12 mg/kg intramuscularly.
Petroleum lubricant is applied to the eyes to protect the animal from corneal injury due to drying.
The animal is shaved from the top of the head to the shoulders and between the ears.
The shaved area is prepped in a sterile fashion first with 7.5% povidone-iodine followed by 70% ethyl alcohol.
The animal is placed onto an operating table with warming blanket and onto a platform that elevates the head and flexes the neck. The head of the animal is directed away from the surgeon.  Adequate flexion of the neck is required in order to adequately expose the occipital ridge.
The animal is draped in a sterile fashion.

### Part 2: Incision and exposure of the bony occiput

Bupivacaine plus epinephrine (Marcaine) 0.25% is applied along the prospective incision site (midline dorsal skin incision along the occiput). This causes local vasoconstriction, limiting bleeding, and provides lasting post-operative analgesia after the animal wakes.
Midline dorsal skin incision is made on the scalp along the occiput.
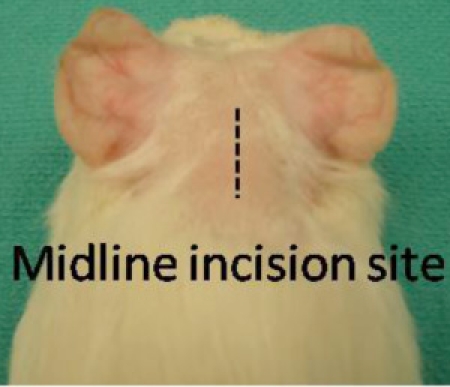
**Figure 1:** Skin incision is made along the dorsal midline, as shown above.
To gain adequate exposure, the skin is retracted laterally by placing a small curved hemostat on each side of the incision.
A horizontal incision is made along the neck muscle to expose the bony occiput.  At this stage the animal may twitch despite adequate anesthesia due to transection of the greater occipital nerve.  Spinal cord transection is a danger with horizontal incision into the suboccipital region.  This complication can be avoided by ensuring that underlying bone is always felt underneath the scalpel blade.
With the suboccipital musculature incised to the bone, a third hemostat is used to retract the superior muscle edge.  This hemostat is held in place by the surgical assistant.
A Lempert periosteal elevator is used to expose the temporo-occipital suture.

### Part 3: Drilling the temporo-occipital suture

Upon exposure of the temporo-occipital suture (Figure 2), an otologic drill with 1.5 mm diamond burr is used to drill inferio-laterally to the suture.
Below the outer table of the skull, the spongy texture of the diploë (The intervening cancellous bone between tables of the skull) will become visible.  Once the diploë has been drilled away, the inner table of the skull appears, along with a faint blue hue that is the sigmoid sinus.
The margins of the sigmoid sinus are defined without penetrating the vessel.
Once the bone overlying the sigmoid sinus has been sufficiently thinned, higher magnification is utilized and a straight pick is used to carefully remove the bone overlying the sigmoid sinus.

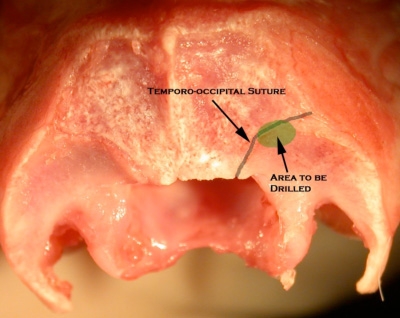
 **Figure 2 **  The area to be drilled partially overlies the temporo-occipital suture.

### Part 4: Obstruction of the Duct

The skeletonized sigmoid sinus is retracted medially to reveal the operculum.
A 0.5 mm diamond burr is used to drill medial to the operculum and into the endolymphatic sac and duct.
The endolymphatic duct is then packed with bone wax using a straight pick.
The skull defect is then reinforced with Gelfoam (Pfizer, New York, NY).
4-0 POLYSORB (United States Surgical, Norwalk, CT) suture is used to bring the muscle together. There is then a seamless transition to a subcutaneous suture technique for closing the skin incision. With this closure there is no need to remove sutures afterward and there is no risk that the animal will claw at the site and pull out a suture.

### Part 5: Post-Operative Care

The guinea pig is put in an incubator to maintain body temperature and checked at 30 minute intervals until awake and able to maintain a sternal position. The animal is inspected for respiration rate and body color.  This center uses enrofloxacin (0.2 mL at 22.7 mg/mL, subcutaneously) and buprenorphine (0.05 mL at 0.3mg/mL, subcutaneously) for post-operative infection prophylaxis and pain control, respectively. Analgesic is given once on the day of surgery and twice on post-operative day one and two. Although antibiotics are not necessary for aseptic surgery, a five-day, twice a day course of prophylactic antibiotics may be givenonce on post-operative day three.

### Complications and Management

Respiratory depression   The surgeon should always be aware of the animal s respirations.  If breathing slows or stops, discontinue surgery and visually inspect for thoracic movement.  If the animal is experiencing respiratory difficulty or labored breathing, check that the mouth and airway are not occluded.
Sigmoid sinus penetration   Use Gelfoam and compression along with the suction tip until bleeding is stopped.
Dural penetration   Cover the defect with Gelfoam.
Vestibular damage   Do not drill more than 1 mm lateral to the sigmoid sinus. Doing so risks injuring the posterior semicircular canal, which would invalidate all data for the guinea pig.

## Discussion

In the weeks following surgical obstruction of the endolymphatic duct, the animal subject experiences fluctuating sensorineural hearing loss as measured by auditory evoked brainstem response. A sample of auditory brainstem response (ABR) recording is shown in Figure 3. The left ear serves as an unoperated control. 
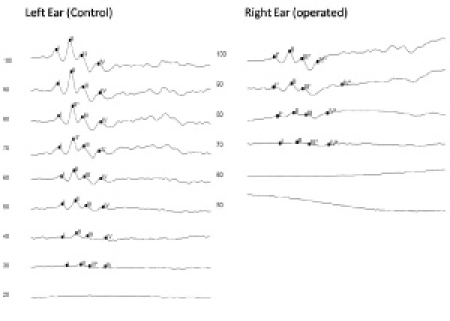
 Click here for a larger figure.**Figure 3: **ABR threshold levels at 16 kHz from a guinea pig 28 weeks post-surgery. The threshold for the left (unoperated) ear is 30 db SPL and for the right (operated) ear is close to 70 dB.

Histological examination of the cochlea also shows distension of Reissner's Membrane (Figure 4).
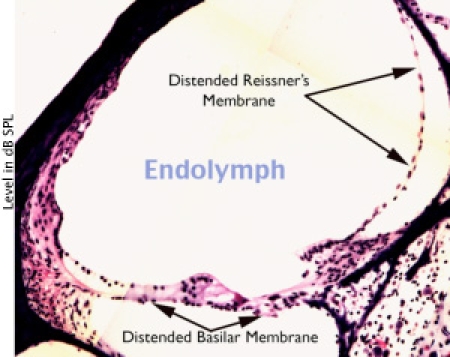
Click here for a larger figure.**Figure 4:** Distension of Reissner s Membrane is a typical finding in ELH.  Measurable distension of the basilar membrane may or may not be present.
